# Cigarette smoking promotes keratinocyte malignancy via generation of cancer stem-like cells

**DOI:** 10.7150/jca.50746

**Published:** 2021-01-01

**Authors:** Shuchun Lin, Wenfeng Mei, Haichun Lai, Xiufeng Li, Huanjiao Weng, Jiani Xiong, Xiuyun Lin, Tao Zeng, Qiong Zhang, Xing Liu, Yunlu Xu, Shubin Fang, Rong Jin, Xiaohua Hu, Jieming Xie, Jianbo Yang, Yiqing Zheng, Yuanzhong Chen, Jizhen Lin

**Affiliations:** 1 The Cancer Center, Fujian Medical University Union Hospital, 29 Xinquan Road, Fuzhou, Fujian 350001, China; 2 Fujian Cancer Hospital & Fujian Medical University Cancer Hospital, 420 Fuma Road, Fuzhou, Fujian 350011, China; 3 The Department of Pharmacology, School of Pharmacy, Fujian Medical University, 1 Xue Yuan Road, University Town, Fuzhou, Fujian 350122, China; 4 The Department of Otolaryngology, Sun Yat-sen Memorial Hospital, Yat-sen Sun University,107 Yan Jiang West Road, Guangzhou, Guangdong 510120, China; 5 The Immunotherapy Research Laboratory, Department of Otolaryngology, Cancer Center, University of Minnesota, 2001 6 th Street SE, Minneapolis 55455, MN, USA

**Keywords:** cigarette smoking, keratinocytes, cancer stem-like cells, octamer-binding transcription factor-4, B lymphoma Mo-MLV insertion region 1 homolog, head and neck squamous cell carcinoma

## Abstract

**Objectives**: Cigarette smoking is involved in the pathogenesis of head and neck squamous cell carcinoma (HNSCC). However, the underlying molecular mechanisms of cigarette smoking-induced HNSCC carcinogenesis are unclear and may involve cancer stem-like cell generation. We examined the effects of cigarette smoke condensate (CSC) on the formation of cancer stem-like cells, which are rich in octamer-binding transcription factor (OCT)-4, inhibitor of differentiation 1 (ID1), nuclear factor (NF)-κB, and B lymphoma Mo-MLV insertion region 1 homolog (BMI-1).

**Materials and Methods**: We used *in vitro*, *in vivo*, and archival human HNSCC tissue analysis to evaluate the effects of CSC on cancer stem-like cell formation.

**Results:** We found that CSC regulated OCT-4 expression, which subsequently regulated ID1 and NF-κB, at the promoter, mRNA, and protein levels *in vitro*. Furthermore, OCT-4 knockdown with siRNA reduced ID1 expression. ID1 and NF-κB synergistically increased the expression of BMI-1 and stimulated keratinocyte sphere generation. *In vivo*, ID1 and NF-κB acted together to generate malignant xenograft tumors, which were aggressive locally and systemically metastatic. Clinical data confirmed that ID1- and NF-κB-positive patients had poor clinical outcomes and 5-year disease-free survival.

**Conclusion**: Our data suggest that smoking cigarettes promoted cancer stem-like cell generation in the head and neck area via the OCT-4/ID1/NF-κB/BMI-1 signaling pathway.

## Introduction

Tobacco use is considered a significant risk factor for the development of head and neck squamous cell carcinoma (HNSCC) 1 and remains a leading risk factor affecting early death and disability worldwide 2. Indeed, the majority of patients with HNSCC report a history of cigarette smoking 3. Statistical analysis has demonstrated that smoking is the most important individual risk factor for many cancers 4 and that cigarette smokers are at a 3-12 times higher risk for developing HNSCC compared with nonsmokers 1. However, how tobacco use is linked to HNSCC is still unclear. Thus, understanding the signaling pathways through which tobacco use promotes HNSCC development is essential for discovering novel potential targets for the treatment of this disease.

Inappropriate activation of developmental transcription factors can stimulate developmental pathways out of context 5. Such changes in developmental transcription factors are related to the generation of stem-like cells that are able to initiate cancer growth. Octamer-binding transcription factor (OCT)-4 is one such transcription factor capable of inducing pluripotent stem cells (iPSCs) from differentiated somatic cells 6, including keratinocytes 7. For example, primary keratinocytes transfected with OCT-4, SRY-box-containing protein 2 (SOX2), Kruppel-like factor 4, and c-MYC exhibit production of iPSCs 7. Moreover, OCT-4 transfection in adult neural stem cells yields embryonic stem-like cells 8. These iPSCs are tumorigenic and cause teratoma formation. Thus, OCT-4 may participate in the generation of immature cells from differentiated keratinocytes in HNSCC patients.

We recently demonstrated that inhibitor of differentiation 1 (ID1) contributes to the tumorigenesis of keratinocytes via regulation of survivin and the phosphatidylinositol 3-kinase (PI3K)/Akt signaling pathways 9. Alternatively, high survivin and PI3K/Akt activity is also observed in cancer and embryonic stem cells 10. However, ID1 alone does not cause metastasis 9.

Nuclear factor kappa-light-chain-enhancer of activated B cells (NF-κB) has been implicated in control of cell proliferation and oncogenesis in many cancers and, as such, has been identified as a therapeutic target 11. Based on the contribution of ID1 to the dedifferentiation of somatic cells 12 and the concomitant effects of NF-κB on the proliferation of cells and induction of epithelial-mesenchymal transition (EMT), it is highly plausible that these pathways may function synergistically in HNSCC development and progression. Thus, we hypothesize that the synergy of ID1 and NK-κB may promote the generation of cancer stem-like cells in keratinocytes.

In this study, we examined whether cigarette smoke condensate (CSC) was associated with HNSCC via induction of OCT-4, and whether such induction promoted the development of HNSCC by increasing the expression of ID1, NF-κB, and the cancer stem cell markers, B lymphoma Mo-MLV insertion region 1 homolog (BMI-1) 13 and CD44 14. We then assessed whether these signaling pathways affected the development and progression of HNSCC in nude mice.

## Material and Methods

### Ethics approval and consent to participate

The surgical samples and clinical data involved in this study were collected according to the IRB at the University of Minnesota and Sun Yat-sen University. The clinical data and materials are available at the Departments of Otolaryngology, Head & Neck Surgery, University of Minnesota and Sun Yat-sen University. The consent form was signed when surgical samples being collected. These clinical data and materials are appropriate for publication.

### Cell lines and HNSCC tissue specimens

CA9-22 cells from oral squamous cell carcinoma were maintained in Roswell Park Memorial Institute 1640 (Life Technologies, Invitrogen, Carlsbad, CA, USA). HOK-16B cells, immortalized keratinocytes, were maintained in keratinocyte basal medium (Lon2a). NA, SCC11A (hypopharynx cancer), and Rhek-1A cells (immortalized keratinocytes) were maintained in minimum Eagle's medium as previously described 9. Cell cultures at 70% confluence were treated with 0.4, 2.0, or 4.0 mg/mL cigarette smoking condensate (CSC) for 12, 24, or 48 h to induce *OCT-4* promoter activity. CSC is a 4% solution (4.0 mg/mL) purchased from Murth Pharmaceuticals (Lexington, Kentucky). The CSC was diluted into 1x (0.4 mg/mL), 5x (2 mg/mL), and 10x (4 mg/mL) solution with dimethyl sulfoxide (DMSO) and the aliquots were stored at -80^o^C. Dihydromethysticin (DHM), purchased from BioCrick (cas# 19902-91-1), was dissolved in ethyl acetate at a concentration of 10 mg/mL for animal gavage.

Fifty-five HNSCC specimens from the Department of Otolaryngology, Sun Yat-sen University (Supplementary Table S1) were used. Twenty-two HNSCC specimens and 12 control tissues (obtained from regions near HNSCC tissues) from the Department of Otolaryngology, University of Minnesota Hospitals and Clinics were used after obtaining written informed consent from patients for research purposes. All specimens and clinical data in this study were procured, handled, and maintained according to the protocols approved by each Institutional Review Board (IRB#1111A07101).

### Induction of xenograft tumors in nude mice with Rhek-IP cells

Cells stably transduced with an empty vector, ID1, NF-κB (p65), or ID1+NF-κB p65 (IP) for up to 6 months were sorted using a FACSAria cell sorter (BD Biosciences). Then, cells expressing high levels of green fluorescent protein were selected and expanded in culture. Athymic nude mice (approximately 16-18 g, n = 6/group) were subcutaneously injected with 1 × 10^6^ cells in their bilateral flanks. After injection, tumor volumes were measured weekly for up to 24 weeks (average: 23.4 weeks). Xenograft tumors in nude mice were harvested, and their sizes, volumes, and weights were measured. Luciferase-positive xenografts were detected with a bioluminescence detector (Xenogen, IVIS; Caliper Life Sciences, Alameda, CA) using standard protocols. Similarly, OTC-4-transduced cells and CD44-positive cells were injected into nude mice to test whether molecules up- and downstream of ID1 and p65 promoted the growth of xenograft tumors. Animal experiments were performed according to a protocol approved by the Institutional Animal Care and Utilization Committee (IACUC ID# 1402-31329A).

### Immunohistochemistry

Tissues were fixed in 10% formalin, cut to a thickness of 4 μm, deparaffinized, and incubated for 90 min with anti-ID1 antibodies (Santa Cruz Biotechnology, Santa Cruz, CA, USA; 1:500 dilution; cat. no. sc-488), anti-p65 antibodies (Santa Cruz Biotechnology; 1:200 dilution; cat. no. sc-109), and anti-OCT-4 antibodies (Abcam, Cambridge, UK; rabbit polyclonal; cat. no. ab19857; 1:250 dilution), as previously described 15. ID1, NF-κB, and OCT-4 immunohistochemistry was performed on HNSCC tissue specimens using standardized protocols.

### Fluorescent-assisted cell sorting (FACS) analysis

Cell cultures (60% confluence) were transduced with an empty vector, ID1, NF-κB, or IP at 1.4 μg/mL for 16 h, recovered in cell culture medium for 24 h, and then harvested for evaluation of positive cells on day 4. Briefly, cells were incubated with anti-BMI-1 antibodies (1:100 dilution), anti-CD44 antibodies (Abcam; cat. no. ab51037; 1:100 dilution), and anti-matrix metalloproteinase (MMP)-9 antibodies (Sigma-Aldrich, St. Louis, MO, USA; 1:100 dilution), incubated at 37 °C for 30 min with fluorescein isothiocyanate-conjugated secondary antibodies, and analyzed on a FACSCalibur instrument using CellQuest Pro (BD Sciences). Cell cycle progression after transfection with the empty vector or OCT-4 was analyzed via flow cytometry, as previously described 9.

### Luciferase assays

Construction of the ID1 reporter was performed as follows: the sequence for the human *ID1* promoter (-1,000 to -1,024 bp including both the *Kpn*I endonuclease site at the 5′-end and the* Hin*dIII endonuclease site at the 3′-end) was amplified from human genomic DNA by polymerase chain reaction (PCR) using the following primer pair: 5′-atggccGGTACCgaccagtttgtcgtctccatggcg-3′ and 5′-gacaagctgtggctccgcactctcAAGCTTggcgag-3′. The PCR-amplified product was subcloned into pGL4 vectors (Promega, Madison, WI, USA) according to the manufacturer's instructions. *CD44* and *MMP-9* reporters were constructed using a method similar to that described above. The *NF-κB* and *OCT-4* promoters were gifts from Dr. Frank Ondrey at the University of Minnesota.

Cells were transduced the next day with the empty vector or OCT-4 plasmids at 1.4 μg/mL and then cotransduced with ID1, NF-κB, CD44, and MMP-9 reporters at 1.4 μg/mL for 16 h in transfection medium. A β-galactocidase reporter was used as a control for transfection efficiency. Cells were harvested for luciferase assays, as previously described 9.

### Reverse transcription (RT)-PCR and quantitative PCR (qPCR)

Cells were cultured in T25-flasks at an initial density of 5 × 10^5^ cells/flask, transfected with an empty vector or OCT-4 for 16 h, recovered in culture medium for 24 h, and harvested for RT-PCR and qPCR. Briefly, total RNA was isolated from the above harvested cells using an RNA Miniprep Kit (Stratagene). The primers used for PCR were as follows: *MMP-9*, 5′-gacacctctgccctcaccat-3′ and 5′-caaaggcgtc gtcaatcacc-3′; *MMP-3*, 5′-atgttaggagaaaggacagtgg-3′ and 5′-ttgg ctgagtgaaagagacc-3′; *ID1*, 5′-ggctgcctgccctgctggac-3′ and 5′-cgccctctcctcgccagtgc-3′; *CD44*, 5′-ggagaaaaatggtcgctaca-3′ and 5′-ggacatagcgggtgccatca-3′; *OCT-4*, 5′-gaggagtcccaggacat caa-3′ and 5′-acactcggaccacatccttc-3′; *SOX2*, 5′-agaaccccaagatgca caac-3′ and 5′-atgtaggtctgcgagctggt-3′; *Nanog*, 5′-ttccttcctccatgg atctg-3′ and 5′-attgttccaggtctggttgc-3′; *BMI-1*, 5′-cttggctcgcattc attttc-3′ and 5′-tcacctcctccttagatttc-3′; and β-actin, 5′-agcaagaga ggcatcctcaccctgaagtac-3′ and 5′-gcacagcttctccttaatgtcacgc acgat-3′. RT-PCR and qPCR protocols were performed as described previously 15.

### Statistical analysis

Student's *t*-tests were used for evaluation of differences between controls and experimental conditions* in vitro*. The Kaplan-Meier survival test was used for evaluation of disease-free survival times according to ID1 and p65 protein expression status. Results with two-sided *p-*values of less than 0.05 were considered significant.

## Results

### OCT-4 was extensively expressed in HNSCC cell lines and HNSCC specimens in association with ID1 and CD44

**Figure 1:**
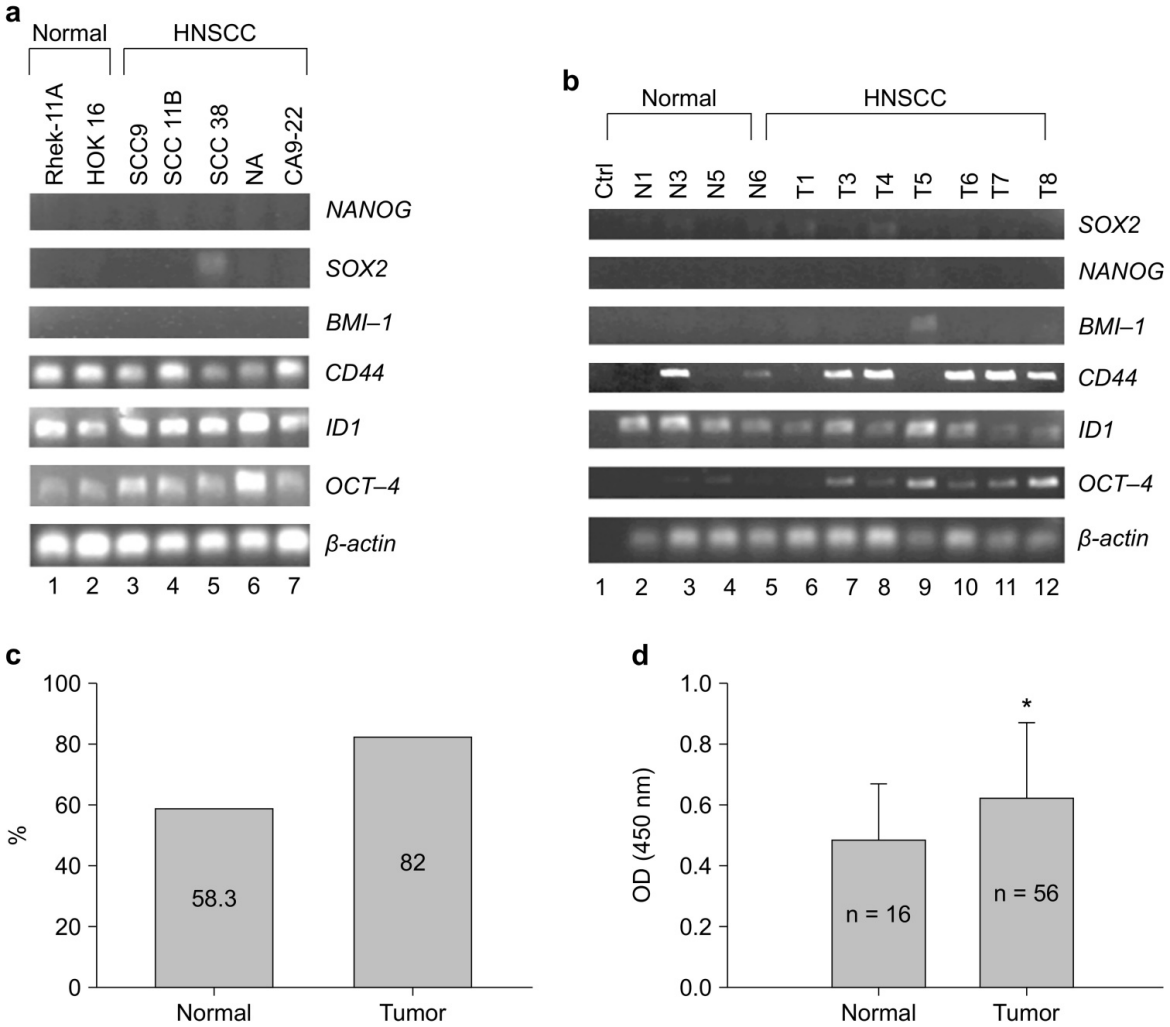
HNSCC cell lines and HNSCC specimens expressed OCT-4. (**A**) Expression of *OCT-4* mRNA in HNSCC cell lines (lanes 3-7) and noncancerous cell lines (lanes 1-2), as determined by RT-PCR. (**B**) Expression of *OCT-4* mRNA in normal (lanes 2-5) and HNSCC (lanes 6-12) tissue samples. (**C**) OCT-4 protein was positive in seven of the 12 (58.3%) normal controls and in 18 of the 22 (82%) HNSCC tumor tissues as determined by immunohistochemistry. The different cellular expression patterns of OCT-4 in HNSCC and control tissues are shown in Figure S2. (**D**) Expression of OCT-4 was evaluated in HNSCC surgical specimens and matched control tissues by ELISA (*p < 0.01). Note that other stem cell markers (Sox2, Nanog, and BMI-1) were rarely expressed in HNSCC tissue samples.

CSC regulated OCT-4 expression in Rhek-1A and CA9-22 cell lines *in vitro* (Supplementary Figure S1). To evaluate whether OCT-4 was expressed in HNSCC, 22 clinical specimens and five HNSCC cell lines were analyzed via RT-PCR, immunohistochemistry, and enzyme-linked immunosorbent assays (ELISAs). Notably, *OCT-4* mRNA transcripts were highly expressed in HNSCC cell lines and weakly expressed in noncancerous cell lines (Figure 1A). Similarly, *OCT-4* mRNA was detected in clinical HNSCC specimens but was absent in normal tissues (Figure 1B). Immunohistochemistry showed that 18 of 22 HNSCC specimens (82.0%) were positive for nuclear OCT-4 (active form), whereas seven of 12 normal tissues (58.3%) were positive for cytosolic OCT-4 (inactive form, Figure 1C). Moreover, OCT-4 was found to be active in the nuclei of HNSCC cells, yet inactive in the cytosol of normal control cells (Supplementary Figure S2). OCT-4 protein was significantly upregulated in HNSCC tissues compared to control tissues, as determined by ELISA (Figure 1D).

### OCT-4 increased the promoter activities of ID1 and NF-κB in immortalized keratinocytes and HNSCC cell lines

**Figure 2:**
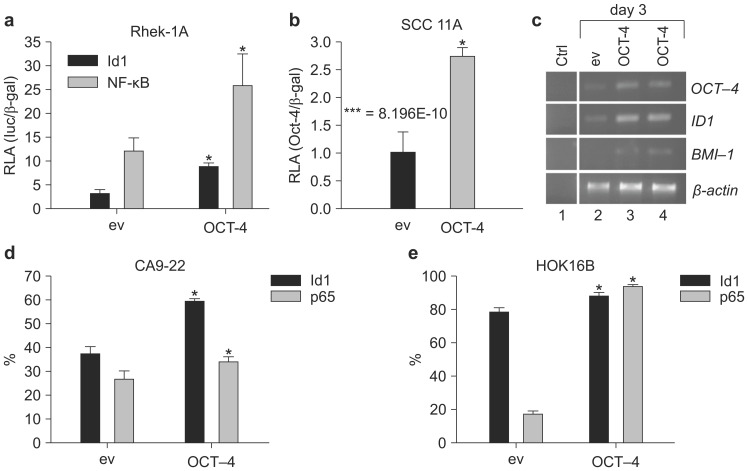
OCT-4 regulated the expression of ID1 and NF-κB in Rhek-1A and HNSCC cells. (**A, B**) The promoter activities of *ID1* and *NF-κB* in Rhek-1A and SCC11A cells following transfection with OCT-4 (*p < 0.05). (**C**) The levels of *ID1* and *BMI-1* mRNA transcripts in Rhek-1A cells after transfection with OCT-4. (**D, E**) The proportion of ID1- and NF-κB (p65)-positive cells in OCT-4-transfected CA9-22 and HOK16B cells compared with those in empty vector (ev)-transfected cells, as evaluated by FACS in triplicate. *p < 0.05.

*OCT-4* cDNA and an *ID1* reporter gene were constructed to evaluate the effects of OCT-4 on ID1. OCT-4 transfection in Rhek-1A cells increased the transcription of *OCT-4* compared to cells transfected with the empty vector (Supplementary Figure S3). Additionally, OCT-4 transient transfection in Rhek-1A and SCC11A cells significantly increased the promoter activity of ID1 compared to cells transfected with empty vector (Figure 2A, B).

Next, to examine whether *ID1* mRNA was upregulated, RT-PCR was performed on 3-day cultures after transient transfection. OCT-4 transfection in Rhek-1A cells increased the expression of *ID1* mRNA transcripts, which was associated with an increase in *BMI-1* transcription (Figure 2C). FACS analysis showed that OCT-4 transfection for 4 days significantly increased the proportions of ID1- and NF-κB-positive CA9-22 cells (Figure 2D) and HOK16B cells (Figure 2E; *p* < 0.05).

### OCT-4 and ID1 regulated CD44 expression in Rhek-1A and HNSCC cells

To examine whether OCT-4 regulated CD44, an HNSCC stem cell marker, luciferase assays were performed. OCT-4 significantly increased the promoter activity and mRNA expression of *CD44* in Rhek-1A, CA9-22, and NA cells (Supplementary Figure S4a). Similar results were observed in Rhek-1A cells by qPCR (Supplementary Figure S4b). ID1 significantly increased the promoter activity and expression of *CD44* in cells, as demonstrated by luciferase assays and FACS analysis, respectively (Supplementary Figure S4c).

### Upregulation of CD44 increased the growth of xenograft tumors in nude mice

CA9-22 cells were used to screen several natural products in our laboratory. Treatment with dihydromethysticin (DHM), a stimulator of CD44-positive cells, increased the number of CD44-positive CA9-22 cells (data not shown). Moreover, administration of DHM (2 mg/mouse/day) by oral gavage for 29 days significantly increased the weights of CA9-22 subcutaneous xenograft tumors in nude mice (Supplementary Figure S4d).

### OCT-4 induced sphere formation

Cell sphere formation is an important characteristic feature of stem cells. To examine whether OCT-4 was involved in the formation of spheres, Rhek-1A cells were transfected with OCT-4, and the number of spheres in cultures and cell cycle progression were evaluated. OCT-4 increased the number of stem-like cells (Supplementary Figure S5a, S5b and S5c). Furthermore, OCT-4-expressing Rhek-1A spheres grew rapidly and reached confluence on day 3. In contrast, empty vector-transfected Rhek-1A spheres grew slowly and remained unattached on day 3 in petri dishes (Supplementary Figure S5d). Lastly, OCT-4-transfected NA cells showed significantly greater proliferation compared with empty vector-transfected cells, as determined by MTT assays (Supplementary Figure S5e).

### OCT-4 siRNA inhibited the expression of ID1 in NA cells

**Figure 3:**
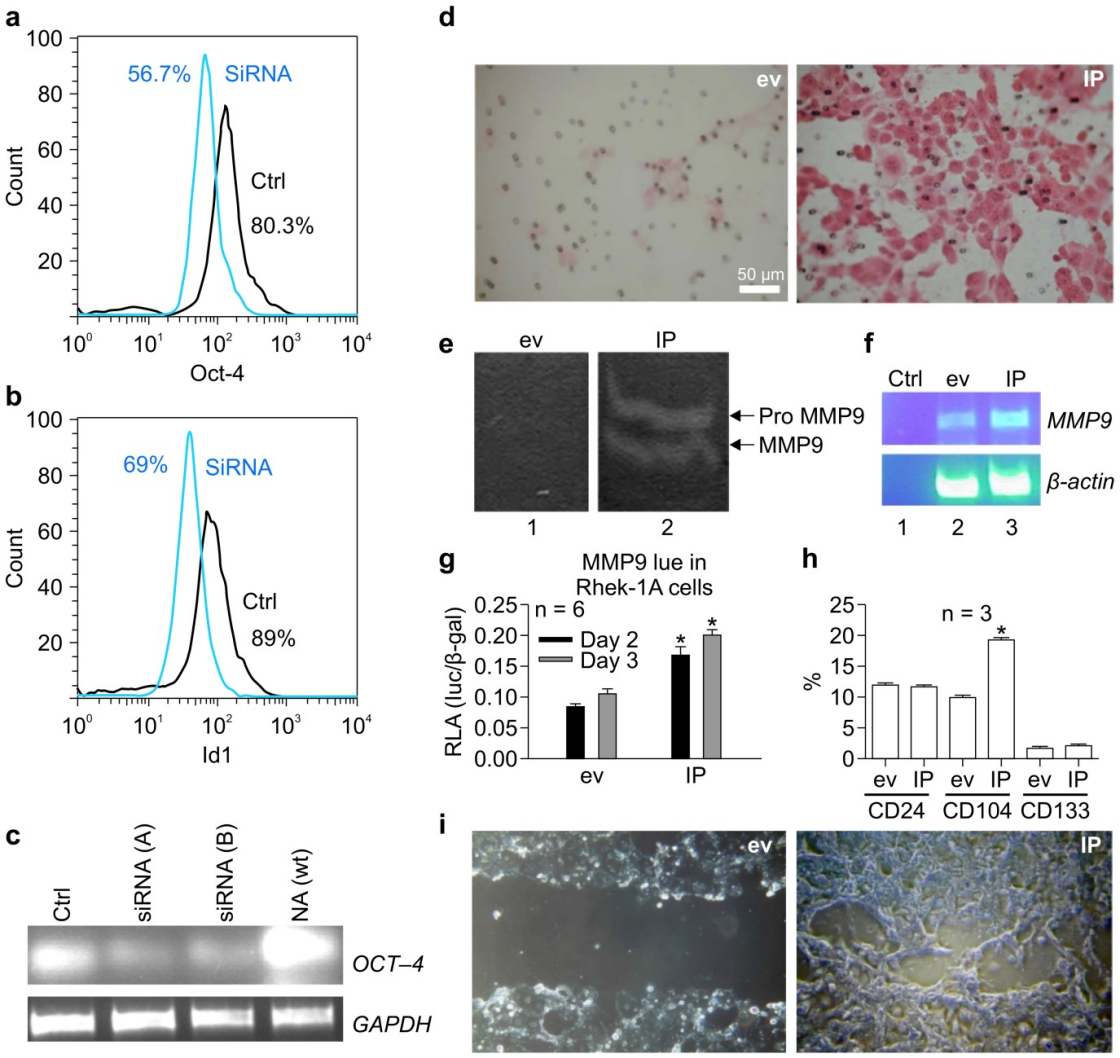
siRNA targeting OCT-4 knocked down ID1 in NA cells. (**A**) Effects of OCT-4 siRNA on the proportion of OCT-4-positive NA cells. (**B**) Effects of OCT-4 siRNA on the proportion of ID1-positive NA cells. (**C**) RT-PCR of *OCT-4* mRNA transcripts in NA cells after stable transfection with siRNAs against OCT-4. ID1 and NF-κB increased the migration of keratinocytes and activity of matrix metalloproteinase (MMP) 9 and BMI-1* in vitro*. (**D**) The migratory activity of keratinocytes promoted by IP in Matrigel. (**E**) Effects of IP on the activity of MMP9. (**F**) Effects of IP on *MMP9* mRNA expression. (**G**) Effects of IP on the promoter activity of MMP9 on days 2-3, as determined by luciferase assays (*p < 0.05). (**H**) Effects of IP transfection on the expression of CD24, CD133, and CD104 by FACS. (**I**) Effects of IP transfection on closure of the scratch compared to cells transfected with empty vector (ev).

To verify that OCT-4 regulated ID1 expression, OCT-4 was knocked down using siRNA in NA cells. OCT-4 siRNA reduced the proportion of OCT-4-positive NA cells from 80.3% to 56.7% (Figure 3A) and simultaneously reduced the proportion of ID1-positive cells from 89% to 69% (Figure 3B). RT-PCR confirmed that siRNA (A-B) specifically knocked down *OCT-4* mRNA transcripts in NA cells (Figure 3C).

### IP synergistically induced the migration of keratinocytes and activity of MMP-9 and BMI-1 in vitro

To determine whether IP regulated the migration of keratinocytes, Matrigel assays were performed. Notably, IP markedly increased the migratory activity of keratinocytes in Matrigel compared to cells transfected with the empty vector (Figure 3D). To evaluate whether this process was associated with an increase in MMP-9 activity, gelatin zymography was performed. We found that IP enhanced MMP-9 activity compared to cells transfected with the empty vector (Figure 3E), and this phenotype was associated with an increase in *MMP-9* mRNA (Figure 3F). Furthermore, luciferase assays demonstrated that IP significantly increased the promoter activity of *MMP-9* on days 2-3 compared to cells transfected with empty vector (Figure 3G). FACS analysis revealed that IP upregulated CD104, yet did not alter CD24 and CD133 expression (Figure 3H). In addition, analysis of cell migration on chamber slides using scratch assays demonstrated that transient transfection with IP for 30 h resulted in nearly full closure of the cell monolayer scratch compared to cells transfected with an empty vector (Figure 3I).

### ID1 and NF-κB p65 synergistically induced the generation of naïve keratinocyte spheres in vitro and metastatic xenograft tumors in nude mice

**Figure 4:**
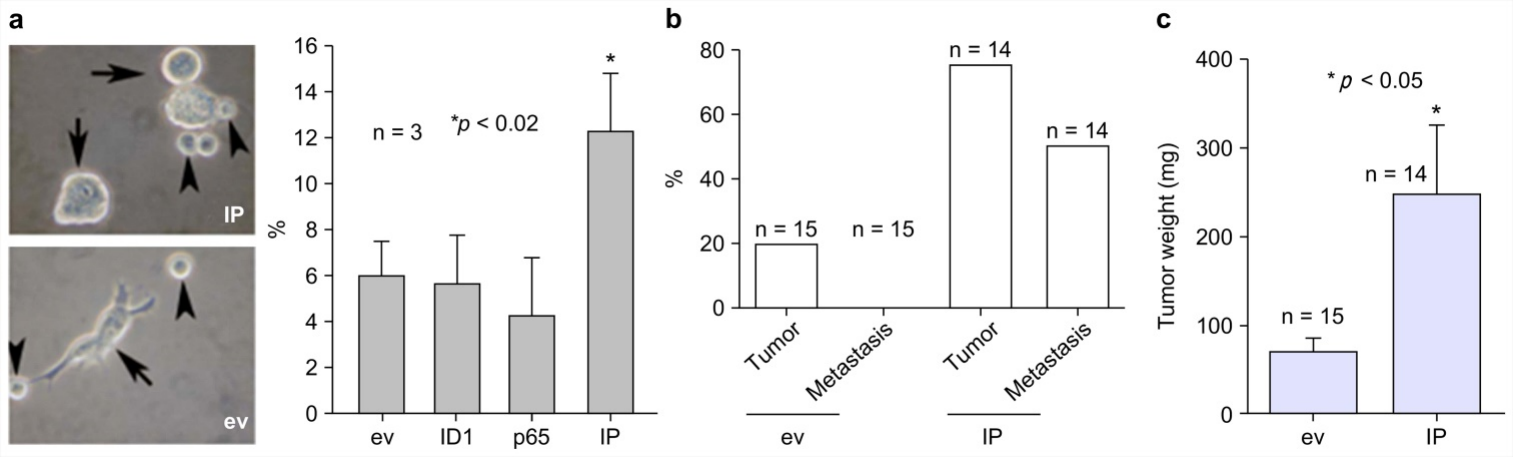
IP induced the growth of aggressive xenograft tumors in nude mice. (**A**) The formation of large keratinocyte spheres in Rhek-1A cells and the proportion of BMI-1-positive cells after transfection with ev, ID1, NF-κB, and IP, respectively. (**B**) Percentages of mice with tumor growth after inoculation of ev-transfected Rhek-1A cells or IP-transfected Rhek-1A cells. (**C**) Tumor weights (*p < 0.05).

To assess the importance of IP in the generation of naïve keratinocyte spheres, IP was stably transfected into Rhek-1A cells for evaluation of their synergistic effects on the formation of cellular spheres, the expression of self-renewal markers, and the growth of tumors in animal models. The results showed that IP synergistically increased the formation of keratinocyte spheres in Rhek-1A cells compared to cells transfected with empty vector (Figure 4A, left panel). FACS analysis showed that IP significantly increased the proportion of BMI-1-positive cells compared to cells transfected with the empty vector, ID1, or NF-κB (Figure 4A, right panel). Moreover, Rhek-1A cells stably transfected with IP triggered the aggressive growth of xenograft tumors in nude mice compared with cells transfected with empty vector in terms of tumor weight (Figure 4B). Representative xenograft tumors induced by IP, ID1, NF-κB, and empty vector are shown in Supplementary Figure S6a. Among 14 nude mice injected with IP-transfected Rhek-1A cells, 11 (78.57%) developed xenograft tumors (average tumor volume: 120.01 ± 105.77 mm^2^). In contrast, only three of 15 (20.0%) xenograft tumors developed in control mice (average tumor volume: 32.0 ± 14.95 mm^2^; Figure 4C).

To visualize the metastatic process, Rhek-1A cells were stably transfected with ID1 and NF-κB and simultaneously labeled with bioluminescence (luciferase). The cells were then injected into the flanks of four nude mice. The results showed that IP-transfected cells grew xenograft tumors in three of the four nude mice, and two of these mice developed metastatic tumors and malnutrition (Supplementary Figure S6b).

### Expression of ID1 and NF-κB in patients with HNSCC was associated with poor clinical outcomes

To verify the importance of IP expression in the clinical setting, we employed an independent set of 55 HNSCC specimens (Supplementary Table S1), stained for IP expression by immunohistochemistry, and evaluated the correlations between IP expression and clinical parameters/outcomes. Using Log-rank (Mantel-Cox) analysis, we observed significant differences in disease-free survival (χ^2^ = 2.66, *p* = 0.077) and lymphatic node metastasis (*p* < 0.05) between IP^+^ and IP^-^ patients.

## Discussion

In this study, we demonstrated for the first time, that CSC was linked to the expression of OCT-4, BMI-1, and CD44, which are expressed in either immature keratinocytes 16,17 or HNSCC stem-like cells 18. After overexpression of OCT-4, keratinocytes expanded rapidly and formed spheres *in vitro* but were unable to trigger xenograft tumor growth in nude mice. Thus, other signals or cofactors from the cancer stem cell niche that modify or affect transactivation of NF-κB or ID1 are needed to transform keratinocytes. Indeed, CSC alone did not transform keratinocytes, but rather potentiated the transformation when combined with other carcinogens. However, when OCT-4 downstream molecules (e.g., ID1 and NF-κB) were overexpressed, cells were capable of initiating xenograft tumors in nude mice and metastasizing *in vivo*.

Malignancy occurs in non-tumorigenic Rhek-1A cells via two developmental transcription factors (ID1 and NF-κB), resulting in cell dedifferentiation coupled with the expression of BMI-1 and CD44. Consistently, ID1 has been shown to be involved in cell dedifferentiation 9, and NF-κB has been shown to be involved in cell proliferation and EMT 18. Importantly, both ID1 and NF-κB are highly regulated in 65-75% of HNSCC cases with a history of smoking 19. In this study, we found that ID1 and NF-κB were important regulators of stem cell markers, such as BMI-1 and CD44, in keratinocytes. The former is considered a self-renewal marker for many stem cells 20. In contrast, CD44 is a specific HNSCC cancer stem cell marker 14.

Experimentally, the synergistic effects of ID1 and NF-κB expression yielded metastatic xenograft tumors in nude mice. This clearly suggested that smoking cigarettes may promote the generation of cancer stem-like cells in the head and neck area via regulation of multiple stem-like cell markers. Moreover, animal studies demonstrated that within 2 months, cells stably transfected with ID1 and NF-κB caused aggressive growth of xenograft tumors in nude mice and induced severe malnutrition. Furthermore, approximately 50% of animals exhibited wasting syndrome (cachexia). Apparently, the synergistic effects of ID1 and NF-κB ultimately duplicated the clinical process of disease and mimicked the malignant behaviors of HNSCC. Coincidently, approximately 50% of patients with HNSCC suffer from recurrence and metastasis 14.

In addition to the upregulation of CD44 and BMI-1, ID1, and NF-κB synergistically regulated the enzymatic activity of MMP-9, which is highly expressed in HNSCC 21 and may be involved in the metastasis of HNSCC 20. Thus, synergy between ID1 and NF-κB may be responsible for the malignant behaviors of HNSCC. Indeed, OCT-4, CD44, ID1, and NF-κB alone contribute to the carcinogenesis of HNSCC 14. However, none of these targets alone were able to induce metastasis of xenograft tumors in nude mice, as shown in this study and a prior study 9. In this study, OCT-4 was not capable of replacing the combined effects of ID1 and NF-κB owing to its dual functions in keratinocyte dedifferentiation or differentiation.

As shown in this study, ID1 and NF-κB alone induced the growth of xenograft tumors that were limited in size and were nonmetastatic in nature. However, synergy between ID1 and NF-κB induced the aggressive growth of xenograft tumors and promoted metastasis of tumors in nude mice. Hence, ID1 and NF-κB together transformed keratinocytes in such a way that the cells became naïve and metastatic. The metastasis of transformed keratinocytes may be related to the expression of MMP-9, which was found to be synergistically upregulated by ID1 and NF-κB. MMP-9 promotes cell migration by degrading collagens and other extracellular matrix proteins in the matrix. However, owing to the small number of animals and differences between xenograft tumors and* in situ* human tumors, further human studies, especially the concentration of CSC in the patients' blood, are warranted to verify our observations.

In summary, our data suggest that CSC promoted the generation of cancer stem-like cells in the head and neck through OCT-4 signaling. Subsequently, OCT-4-induced synergistic action of ID1 and NF-κB triggers the expression of important head and neck cancer stem cell markers, BMI-1 and CD44.

## Supplementary Material

Supplementary figures and table.

## Abbreviations

HNSCC: head and neck squamous cell carcinoma
CSC: cigarette smoke condensate
OCT: octamer-binding transcription factor
ID1: inhibitor of differentiation 1
NF: nuclear factor
BMI-1: B lymphoma Mo-MLV insertion region 1 homolog
iPSC: inducing pluripotent stem cells
SOX2: SRY-box-containing protein 2
PI3K: phosphatidylinositol 3-kinase
EMT: epithelial-mesenchymal transition
FACS: fluorescent-assisted cell sorting

## References

[1] Blot WJ, McLaughlin JK, Winn DM, Austin DF, Greenberg RS, Preston-Martin S (1988). Smoking and drinking in relation to oral and pharyngeal cancer. Cancer Res.

[2] GBD 2015 Tobacco Collaborators (2017). Smoking prevalence and attributable disease burden in 195 countries and territories, 1990-2015: a systematic analysis from the Global Burden of Disease Study 2015. Lancet.

[3] Gupta B, Johnson NW, Kumar N (2016). Global epidemiology of head and neck cancers: a continuing challenge. Oncology.

[4] Ordóñez-Mena JM, Schöttker B, Mons U, Jenab M, Freisling H, Bueno-de-Mesquita B (2016). Quantification of the smoking-associated cancer risk with rate advancement periods: meta-analysis of individual participant data from cohorts of the CHANCES consortium. BMC Med.

[5] Micalizzi DS, Christensen KL, Jedlicka P, Coletta RD, Barón AE, Harrell JC (2009). The Six1 homeoprotein induces human mammary carcinoma cells to undergo epithelial-mesenchymal transition and metastasis in mice through increasing TGF-beta signaling. J Clin Invest.

[6] Takahashi K, Yamanaka S (2006). Induction of pluripotent stem cells from mouse embryonic and adult fibroblast cultures by defined factors. Cell.

[7] Aasen T, Raya A, Barrero MJ, Garreta E, Consiglio A, Gonzalez F (2008). Efficient and rapid generation of induced pluripotent stem cells from human keratinocytes. Nat Biotechnol.

[8] Kim JB, Sebastiano V, Wu G (2009). Oct-4-induced pluripotency in adult neural stem cells. Cell.

[9] Lin J, Guan Z, Wang C, Feng L, Zheng Y, Granados EC (2010). Id1 contributes to HNSCC survival via the NF-kB/survivin and PI3K/Akt signaling pathways. Clin Cancer Res.

[10] Armstrong L, Hughes O, Yung S, Hyslop L, Stewart R, Wappler I (2006). The role of PI3K/AKT, MAPK/ERK and NF kappa beta signalling in the maintenance of human embryonic stem cell pluripotency and viability highlighted by transcriptional profiling and functional analysis. Hum Mol Genet.

[11] Orlowski RZ, Baldwin AS Jr (2002). NF-kappaB as a therapeutic target in cancer. Trends Mol Med.

[12] Li Y, Yang J, Luo JH, Dedhar S, Liu Y (2007). Tubular epithelial cell dedifferentiation is driven by the helix-loop-helix transcriptional inhibitor Id1. J Am Soc Nephrol.

[13] Molofsky AV, Pardal R, Iwashita T, Park IK, Clarke MF, Morrison SJ (2003). Bmi-1 dependence distinguishes neural stem cell self-renewal from progenitor proliferation. Nature.

[14] Prince ME, Sivanandan R, Kaczorowski A, Wolf GT, Kaplan MJ, Dalerba P (2007). Identification of a subpopulation of cells with cancer stem cell properties in head and neck squamous cell carcinoma. Proc Natl Acad Sci U S A.

[15] Lin J, Tsuprun V, Kawano H, Paparella MM, Zhang Z, Anway R (2001). Characterization of mucins in human middle ear and eustachian tube. Am J Physiol Lung Cell Mol Physiol.

[16] Cordisco S, Maurelli R, Bondanza S, Stefanini M, Zambruno G, Guerra L (2010). Bmi-1 reduction plays a key role in physiological and premature aging of primary human keratinocytes. J Invest Dermatol.

[17] Chiou SH, Yu CC, Huang CY, Lin SC, Liu CJ, Tsai TH (2008). Positive correlations of Oct-4 and Nanog in oral cancer stem-like cells and high-grade oral squamous cell carcinoma. Clin Cancer Res.

[18] Huber MA, Azoitei N, Baumann B, Grünert S, Sommer A, Pehamberger H (2004). NF-kappaB is essential for epithelial-mesenchymal transition and metastasis in a model of breast cancer progression. J Clin Invest.

[19] Lessard J, Sauvageau G (2003). Bmi-1 determines the proliferative capacity of normal and leukaemic stem cells. Nature.

[20] Franchi A, Santucci M, Masini E, Sardi I, Paglierani M, Gallo O (2002). Expression of matrix metalloproteinase 1, matrix metalloproteinase 2, and matrix metalloproteinase 9 in carcinoma of the head and neck. Cancer.

[21] Ruokolainen H, Paakko P, Turpeenniemi-Hujanen T (2004). Expression of matrix metalloproteinase-9 in head and neck squamous cell carcinoma: a potential marker for prognosis. Clin Cancer Res.

