# Supplementary material for: Cigarette smoking promotes keratinocyte malignancy via generation of cancer stem-like cells
Source: J Cancer. 2021 Jan 1;12(4):1085–93. doi: 10.7150/jca.50746 (PMC7797662; doi:10.7150/jca.50746)
Supplement: Supplementary file 1 — Supplementary figures and table. [file jcav12p1085s1.pdf]

Supplementary Material

Supplementary Table S1. Clinical data and related statistical analyses for 55 patients with HNSCC.

Clinicopathological features		n	IP^+	χ^2	*P*
Age	≤ 60	24	10	0.219	0.640
	> 60	31	11		
Sex	Male	44	18	0.693	0.405
	Female	11	3		
Clinical classification	Tongue	10	3	0.781	0.941
	Orapharyngeal	10	4		
	Glottic	10	4		
	Supraglottic	12	4		
	Hypopharyngeal	13	6		
Clinical stage	1+2	15	6	0.029	0.865
	3+4	40	15		
Differentiation	Poor	4	1	0.322	0.851
	Moderate	13	5		
	Well	38	15		
Lymph node metastasis	Negative	26	9	0.266	0.606
	Positive	29	12		
Survival	< 5 years	31	15	3.135	0.077
	≥ 5 years	24	6		
Smoke	Yes	37	15	0.266	0.606
	No	18	6		

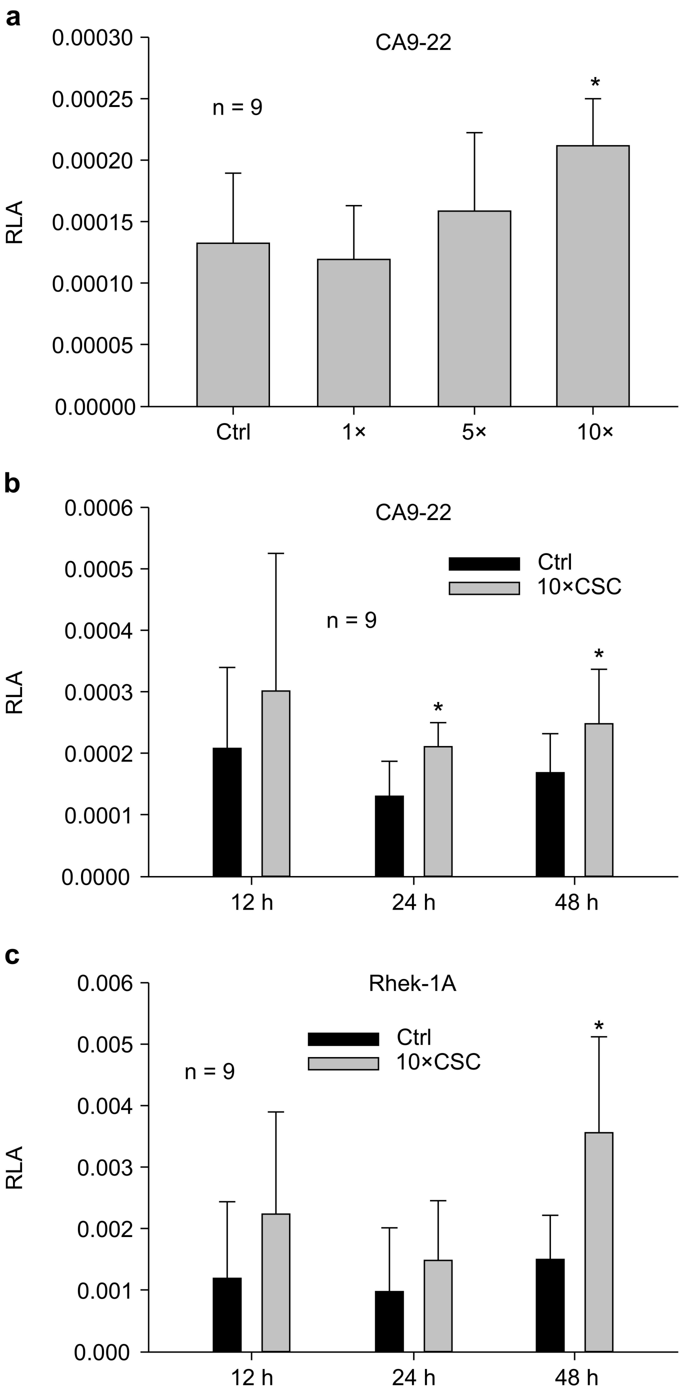

Supplementary Figure S1. Cigarette smoking condensate (CSC) regulates the promoter activity of OCT-4 in Rhek-1A and HNSCC cell lines. (**A, B**) CSC increased the promoter activity of OCT-4 in a concentration- and time-dependent manner in CA9-22 cells. (**C**) CSC increased the promoter activity of OCT-4 in a time-dependent manner in Rhek-1A cells. 10× CSC, equivalent to 4 mg/mL CSC.

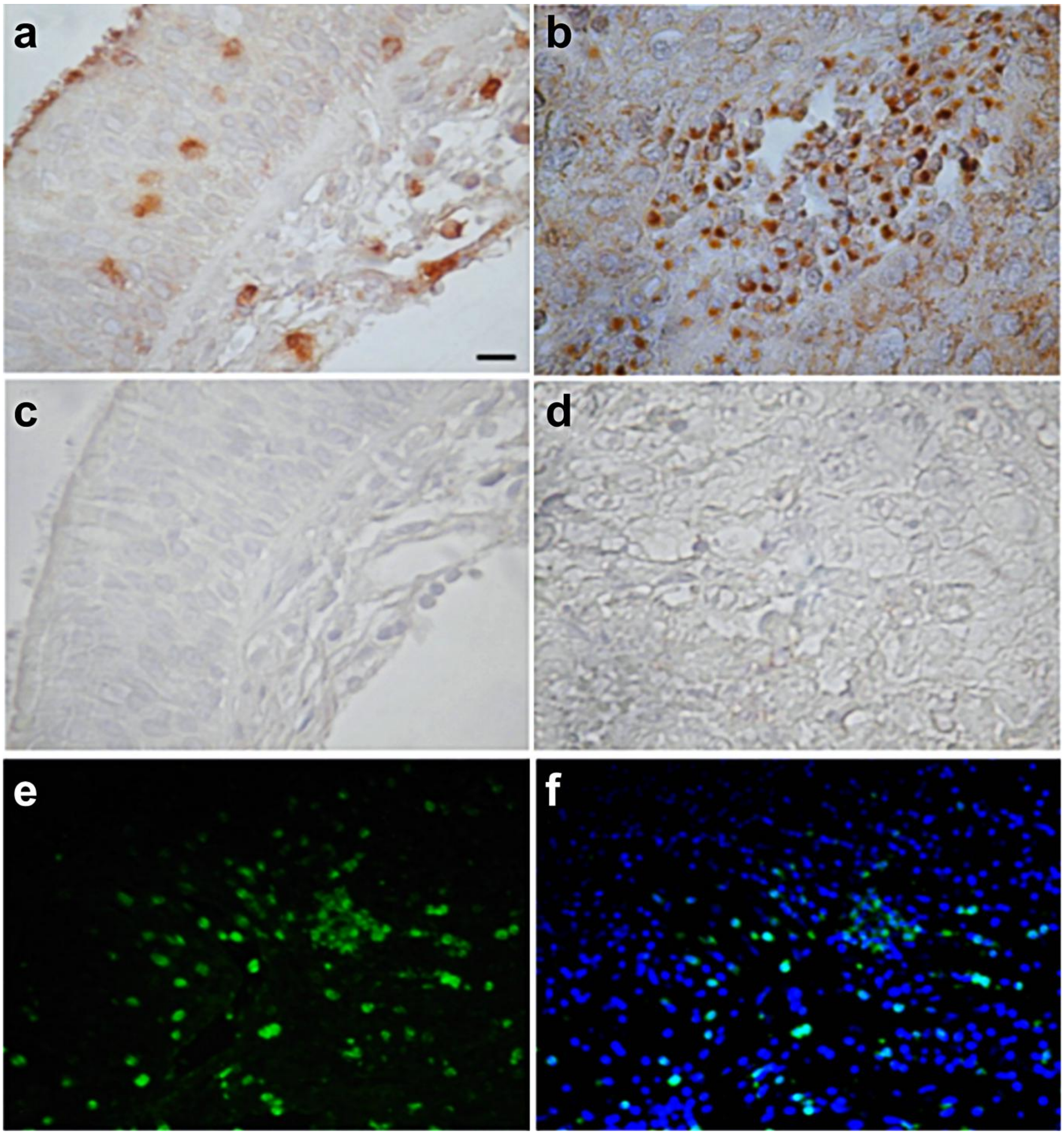

Supplementary Figure S2. Differential patterns between normal and cancerous tissues by immunohistochemistry. (**A**) Approximately 58.3% of normal control tissues exhibited positive staining for OCT-4 in the cytosol, (B) whereas approximately 82.0% of HNSCC tissue specimens stained positively for OCT-4 in the nucleus. Immunohistochemical controls for (**A**) and (**B**) are shown in (**C**) and (**D**), respectively. (**E**) Fluorescent immunohistochemistry demonstrated that OCT-4 was located in the nuclei of tumor cells, which overlapped with the nuclear dye DAPI (**F**). Bar in a = 10 μM (applies to **B**–**F**).

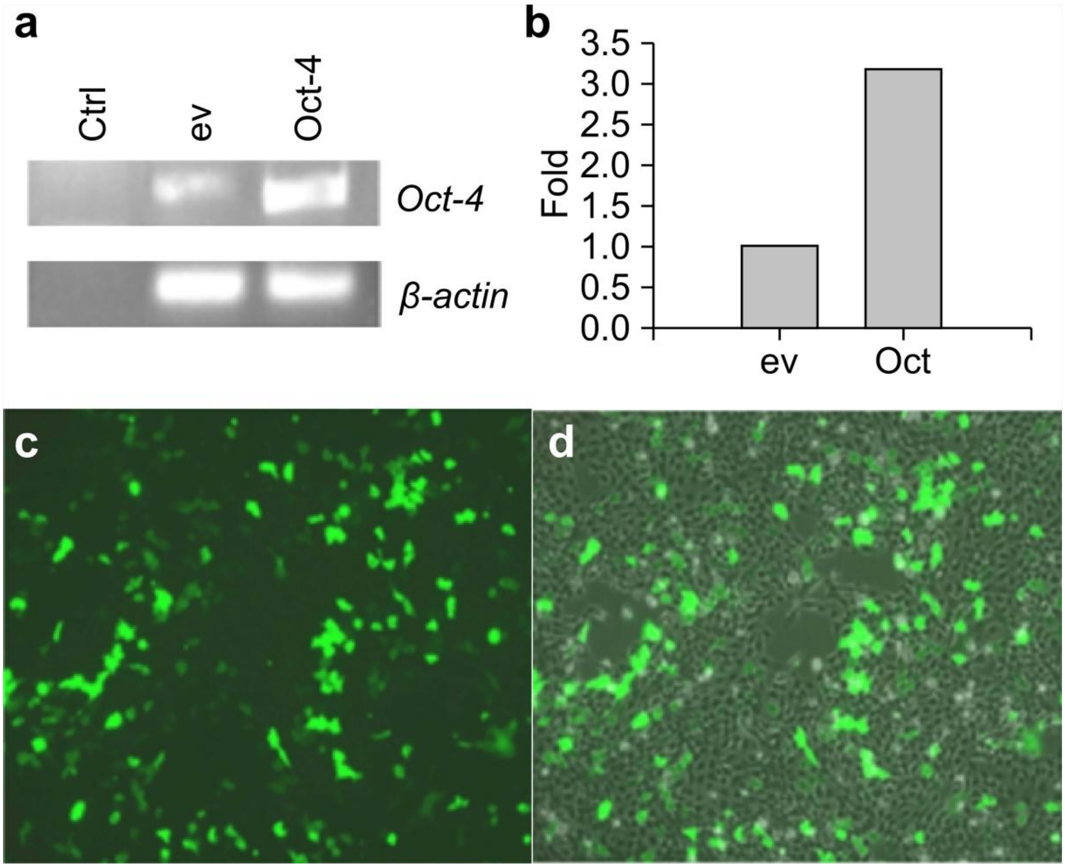

Supplementary Figure S3. (**A**) OCT-4 transfection increased the expression of the *OCT-4* mRNA transcripts in Rhek-1A cells compared with that in cells transfected with empty vector (ev). (**B**) OCT-4-transfected Rhek-1A cells had a greater than 3-fold increase in mRNA expression compared with ev-transfected cells after normalization to β-actin expression. (**C**) Inverted microscopy showed FITC-positive cells (high, moderate, and weak expression) 4 days after transfection. (**D**) FITC-positive cells merged with phase micrographs in the same field, demonstrating the transfection efficiency (approximately 70%).

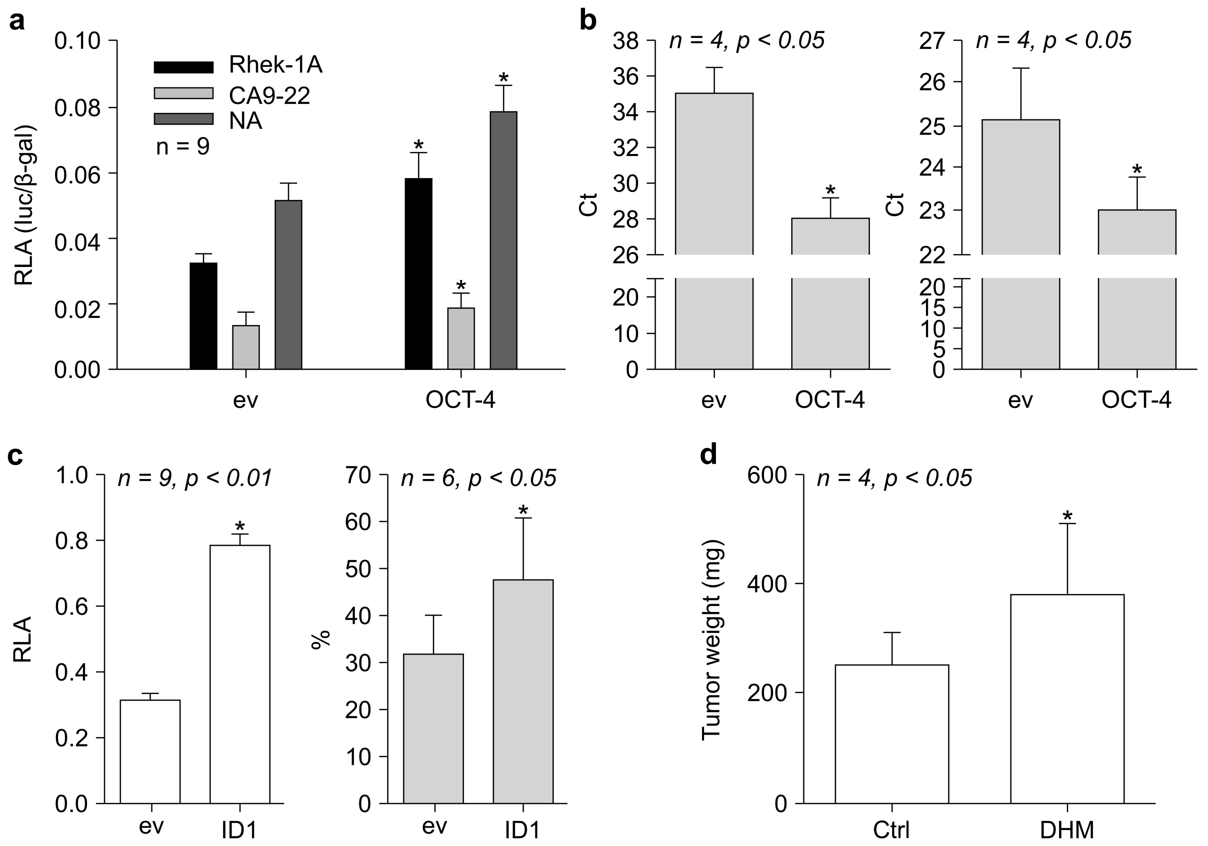

Supplementary Figure S4. OCT-4 regulated the promoter activity of CD44 in Rhek-1A, CA9-22, and NA cells. (**A**) Luciferase assays showing the effects of OCT-4 on the promoter activity of CD44 in Rhek-1A, CA9-22, and NA cells. (**B**) qPCR showing the effects of OCT-4 on *CD44* (left) and *ID1* (right) mRNA levels in Rhek-1A cells (*$P < 0.05$). (**C**) Luciferase assays and FACS analyses showing the effects of ID1 on the promoter activity of CD44 (left) and % of CD44-positive Rhek-1A cells. (**D**) Effects of dihydromethysticin (DHM), a stimulator of CD44-positive cells, on the weights of CA9-22 s.c. xenograft tumors in nude mice after administration at a dose of 2 mg/day/mouse for 29 days via oral dosing gavage (2 mg DHM in 100 μL of polyethylene glycol:alcohol = 9:1, 50 μL per gavage, twice per day).

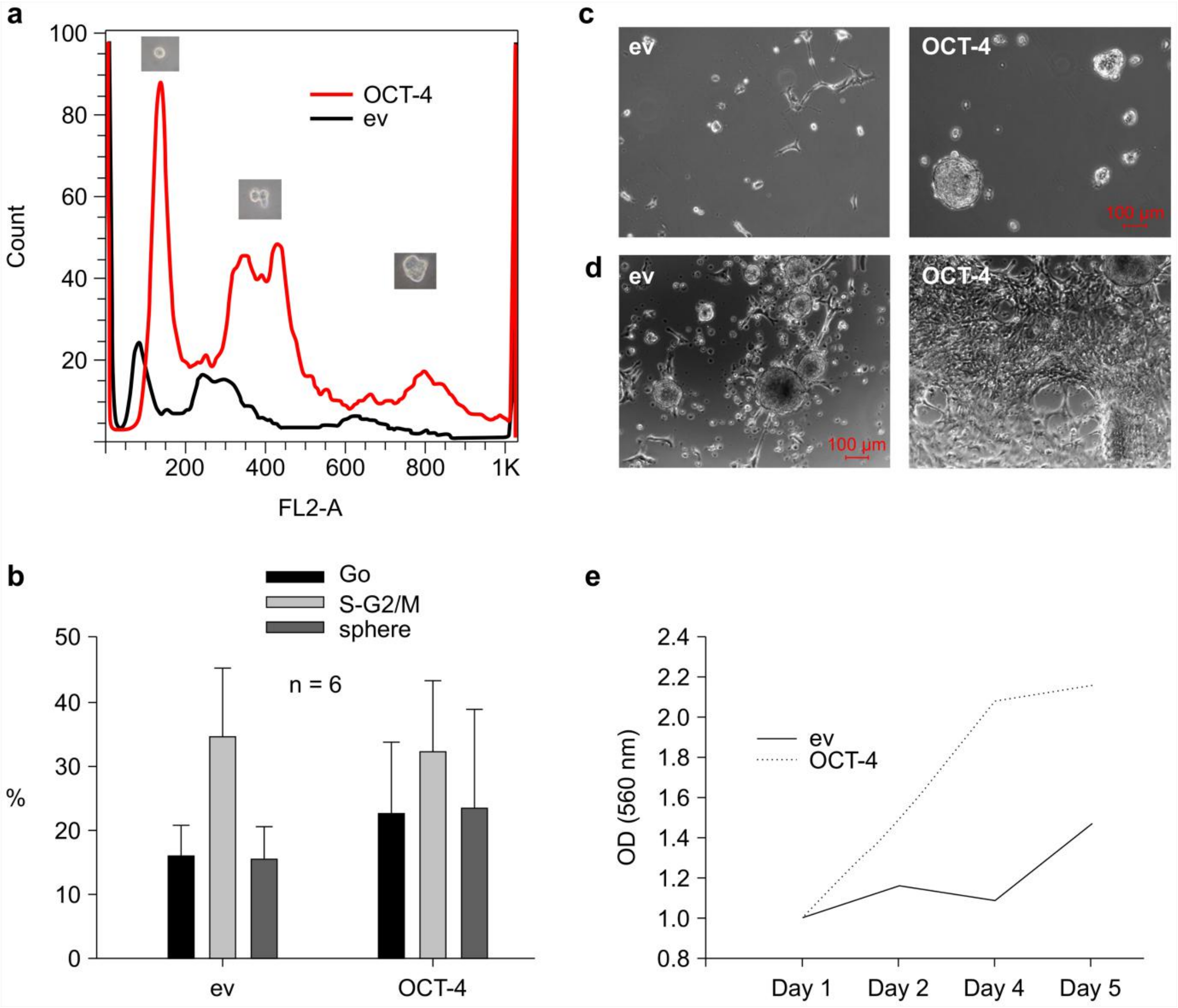

Supplementary Figure S5. Spheres were defined as multinuclear large cells with DNA volume greater than 2-fold. (**A**) OCT-4 transfection in Rhek-1A cells increased cell size compared with ev transfection, as demonstrated by flow cytometry. (**B**) Quantitative analysis of the cell cycle data demonstrated that OCT-4 increased the sphere percentage. (**C**) OCT-4 increased the formation of spheres compared with ev in Rhek-1A cells. (**D**) OCT-4 promoted sphere attachment and spread on day three compared with ev. (**E**) OCT-4 markedly increased cell proliferation in NA cells compared with ev transfection, as demonstrated by MTT.

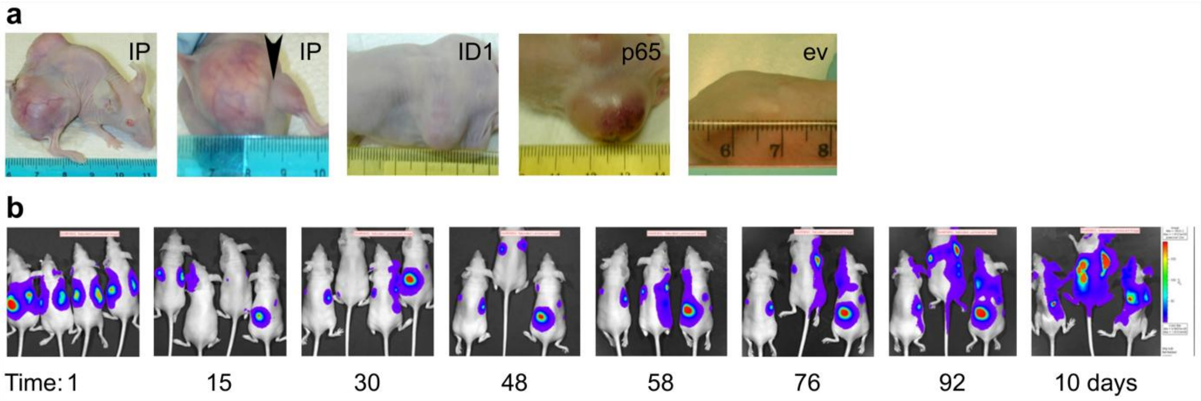

Supplementary Figure S6. IP triggered the aggressive growth of xenograft tumors in nude mice compared with ID1, NF-κB, and ev alone (**A**). Note that IP-induced xenograft tumors invaded into the surrounding tissues, including the leg (arrowhead), from the flanking back skin and caused wasting in nude mice (first and second panels). Bioluminescent imaging demonstrated that IP induced metastatic xenograft tumors in 2 of the 4 mice (**B**). Note that the majority of the injected cells died at day 15 following injection. One mouse did not develop any tumors, one mouse had an original xenograft tumor at the injection site and two mice had original xenograft tumors in the injection sites as well as multiple metastasized foci from day 58 on. Note that there were two mice with wasting status on day 110. Exposure time, 1 min.
